# Time to recovery and its predictors among children aged 6–59 months with severe acute malnutrition admitted to outpatient therapeutic program in Southwest Ethiopia: retrospective cohort study

**DOI:** 10.1186/s12887-022-03205-5

**Published:** 2022-03-26

**Authors:** Smegnew Gichew Wondie, Beakal Zinab, Getu Gizaw, Meseret Tamrat

**Affiliations:** 1 Department of Human Nutrition and Dietetics School of Public Health, Mizan Tepi University, Mizan Aman, South West Ethiopia Ethiopia; 2 Department of Human Nutrition and Dietetics School of Public Health, Jimma University, Jimma, Ethiopia

**Keywords:** Severe acute malnutrition, Outpatient therapeutic program, Recovery time

## Abstract

**Background:**

Outpatient therapeutic program (OTP) brings the services for the management of Severe Acute Malnutrition (SAM) closer to the community by making services available at decentralized treatment points within the primary health care setting. Despite the available interventions to tackle nutritional problems, there is scarce information on time to recovery and its predictors. Therefore, the aim of this study was to estimate time to recovery and identify its predictors among children aged 6–59 month with SAM admitted to OTP in Bench Sheko zone Southwest Ethiopia.

**Methods:**

A retrospective cohort study was conducted on 588 children who had been managed for SAM under OTP, from September 01, 2018, to August 30, 2019, in 4 public health centers in Bench Sheko zone. A total of 1301 children’s card were eligible from them 588 children’s cards were selected by simple random sampling methods. Data was entered into EPI- data version 4.4.2 and exported to SPSS version 20 for analysis. Kaplan Meir estimate median time to recovery and survival curve was used to compare the time to recovery using a log-rank test among different characteristics. Cox Proportional Hazard Model was used to identify significant predictors of time to recovery. Association was summarized by using adjusted hazard ratio (AHR) and statistical significance was declared at 95% CI, and *P*-value < 0.05.

**Result:**

Recovery rate was 54.4% with the median recovery time 49 days with an Interquartile range of 21 days. The independent predictors of nutritional recovery time were: newly admitted (AHR = 1.52, 95% CI: 1.17, 2.98),had no diarrhea (AHR = 1.9, 95% CI: 1.52, 2.42), had no cough (AHR = 1.4, 95% CI: 1.13, 1.74) had no blood stool (AHR = 1.55, 95% CI: 1.14, 2.10) had no malaria (AHR = 1.75, 95% CI: 1.32, 2.32), and took deworming (AHR = 1.4, 95% CI: 1.01–1.61).

**Conclusion and recommendation:**

In the current study recovery rate and the median time of recovery is by far below the standard. Cough, diarrhea, malaria, deworming and admission status were independently associated with recovery time. Health professionals should give attention for early detection and management of co-morbidities. Minster of health should give refreshment community based management of acute malnutrition training for health workers to follow the national guideline strictly.

## Introduction

Adequate nutrition is recognized as a key determinant of health and well-being, and a contributor to human capital development [1, 2]. Optimal physical growth and cognitive development are founded on child nutrition with long-term health and economic implications for individuals and nations [3]. Malnutrition is chronic and lifelong, a highly preventable condition that begins in early childhood and continues into old age, devastating one generation, and passing the miserable legacy on to the next [2, 3].

In 2021 globally, approximately 149.2 million children under 5 suffer from stunting, 45.4 million were wasted and 38.9 million were overweight [1]. Severe acute malnutrition (SAM) affects nearly 20 million children under 5 years, mostly from the African Region and South-East Asia Region causing up to 1 million deaths each year by increasing susceptibility to death from severe infection [4–6].

Studies done in the other area related to this topic indicate the recovery time of severely acutely malnourished children under the OTP is under the internationally accepted standard and The CMAM (community based management of acute malnutrition) program originated based on two important pieces of evidence. First, when severely malnourished children access early nutritional care during the development of their condition and continue in the CMAM program until they have recovered, then there will be high rates of recovery. On the contrary, if severe acute malnourished children enroll in care late and/or they are not encouraged to stay in the program for as long as needed, then success rates of treatment are likely to be low [2, 7].

Second there are variability of predictors and rate of recovery shown by different studies conducted before [8–10]. So it is important to do study in the study area to know the time to recovery and its significant determinants. Therefore this study aims to determine the time to recovery and its predictors with an outpatient treatment program of severe acute malnutrition in the health center of Bench Sheko zone, southern Ethiopia to fill the gap of scientific performance evidence for responsible stakeholders and deliver information on time to recovery and its predictors.

### Significance of the study

Ethiopia is one of the developing countries with a high prevalence of acute malnutrition in the world. Even though delivery of Outpatient therapeutic program play a great role for the management of SAM, multiple evidences indicated significant variability in recovery time thus, context specific understanding time to recovery and its associated factor is vital for giving quality care and provide its contribution on improving the rate of recovery, reduce the death rate, defaulting rate, and non-respondents as a result reduction of long term effects such as socioeconomic loss and poor quality of life.

The finding of this study will be also used as a source of scientific evidence and performance evaluation clinicians and zonal health bureaus and other NGOs who are working on the area.

## Methods and materials

### Study design

A retrospective cohort study was conducted.

### Study setting and period

The study was conducted from February 21/ 2020 to March 20/2020 Bench Sheko zone, Public health institutions which is one of the 25 zonal administrations in the SNNP(South nation nationality of people region) region of Ethiopia, located 562 km away from Addis Ababa (the capital city of Ethiopia). The total population of the zone is estimated to be 625,345 and the number of under-five children in the zone is estimated to be 97,616. Its climatic condition is weina dega, and gets rain most of the time in different seasons per year. Regarding to the health facilities, there are one general teaching hospital, 26 health centers, and 128 health posts. OTP service is delivering only in health center level total 11 health center gives the service.

## Population

### Source population

All records of children aged 6–59 months who were treated on the OTP at health institution of Bench Sheko zone from September 01, 2018, to August 30, 2019.

#### Study population

Records of all randomly selected eligible children aged 6–59 months who were treated on OTP at selected health institution the year between September 01, 2018, and August 30, 2019.

On children’s outpatient therapeutic program registration card health professional filled the information about children’s nutritional status and medical complication like cough, diarrhea, vomiting and fever etc. We were extracted this data retrospectively to assess time to recovery and its predictors among children aged 6–59 months with severe acute malnutrition admitted to outpatient therapeutic program in Southwest Ethiopia (Table 1).

**Table 1:**
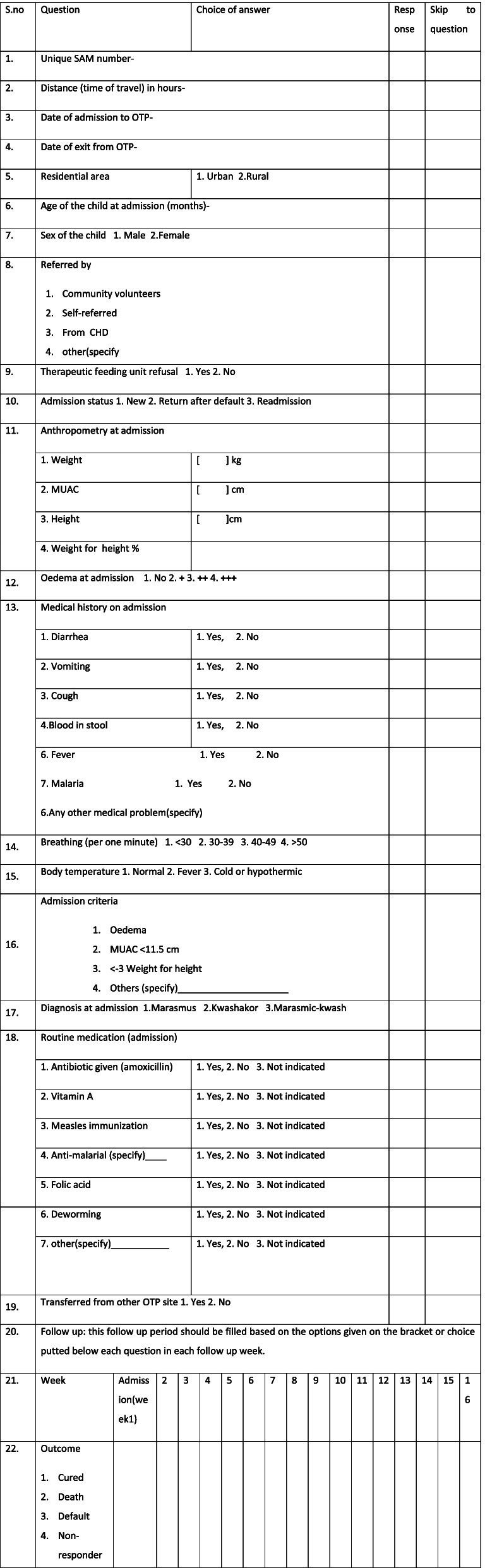
Data extraction tool to assess time to recovery and its predictors among children aged 6–59 months with severe acute malnutrition admitted to outpatient therapeutic program in Southwest Ethiopia

## Eligibility criteria

### Inclusion criteria

All children aged 6–59 months who were admitted to OTP according to national admission criteria from September 01, 2018- August 30, 2019 in Bechi Sheko zone health facility. The admission criteria for OTP according to WHO 2013 SAM management protocol with Mid Upper Arm Circumference (MUAC) of less than 115 mm and /or presence of bilateral pitting edema. Regardless of these, children presented with medical problems won’t be admitted to the OTP. Rather, they need to be referred to as therapeutic feeding units (TFU). SAM Children would discharged from OTP when weight-for-height/length is ≥ − 2 Z-score or MUAC greater than 125 mm. Kwashiorkor cases discharged from the OTP after their edema is disappeared regardless of their body weight status. These children declared as recovered however, children may have different outcomes such as defaulter, non- respondent, medical transfer and died [11].

### Exclusion criteria

Documents with incomplete data that is time of admission or time of exit not recorded were excluding. Those transferred-in and transferred-out children between the study periods were excluded from the study because we couldn’t find full information. According to the data found from selected health centers total admission on the outpatient program between the years September 01, 2018, to August 30, 2019 was 2166. From those 214 were age greater than 59 months, no admission at age less than 6 months, 520 respondents had incomplete data, and transfer in and transfer out cases were 131. Accordingly Out of 2166 respondent cards 1301 cards were eligible for the current study and 865 respondent cards were excluded from the study by using the exclusion criteria. Total eligible admission cards were listed in selected health centers.

### Sample size determination and sampling procedure

#### Sample size calculation by using outcome variable

The sample size was determined by using STATA 14 software the following assumption was considered, 95% confidence level, power 80%, design effect 1.5 and 1.5 adjusted hazard ratio to be detected as significant (equivalence of medium effect size) for time-to-recovery of the outcome variable [12]. Different studies regarding the same design and topic were revised to calculate sample size and finally, the largest sample size was taken among them as summarized (Table 2).

**Table 2:** Sample size calculation by using STATA 14 software after searching different related literature for of recovery rate among sever acutely malnourished 6–59 months of children treated with an outpatient therapeutic program in Bench sheko zone from September 01, 2018 to August 30, 2019

NO	Reference	PE	PC	AHR	N	Design effect	Total sample size
1.	[13]	0.62	0.38	1.5	392	1.5	588
2.	[14]	0.65	0.35	1.5	357	1.5	536
3.	[15]	0.78	0.22	1.5	248	1.5	449

*PE probability of an event

*PC probability of censored

*CI confidence interval 95%

*power 80%

*AHR adjusted hazard ratio

#### Sample size determination by using predictors

The sample size determination for determinants of treatment recovery time of OTP is calculated by STATA version 14.1. After reviewing different kinds of literature and use the most determinant factor with the largest sample size as summarized (Table 3).

**Table 3:** Sample size calculation by searching determinants from different related literature for the predictor of recovery time among sever acutely malnourished 6–59 months of children treated with an outpatient therapeutic program in Bench sheko zone from September 01, 2018, to August 30, 2019

No	Factors	CI	AHR	Power	PE	PC	Sample size	Design effect	sample size	Reference
1.	Distance from home to HI < 2 h	95	1.48	80	0.81	0.19	312	1.5	468	[10]
2.	Amoxicillin intake at admission	95	1.95	80	0.721	0. 28	136	1.5	204	[9]
3.	Admission W/H > 60%	95	1.87	80	0.784	0.22	131	1.5	197	[10]

*PE probability of an event

*PC probability of censored

*CI confidence interval 95%

*power 80%

*AHR adjusted hazard ratio

According to Tables 1 and 2 the largest sample size was 588.

**Fig. 1:**
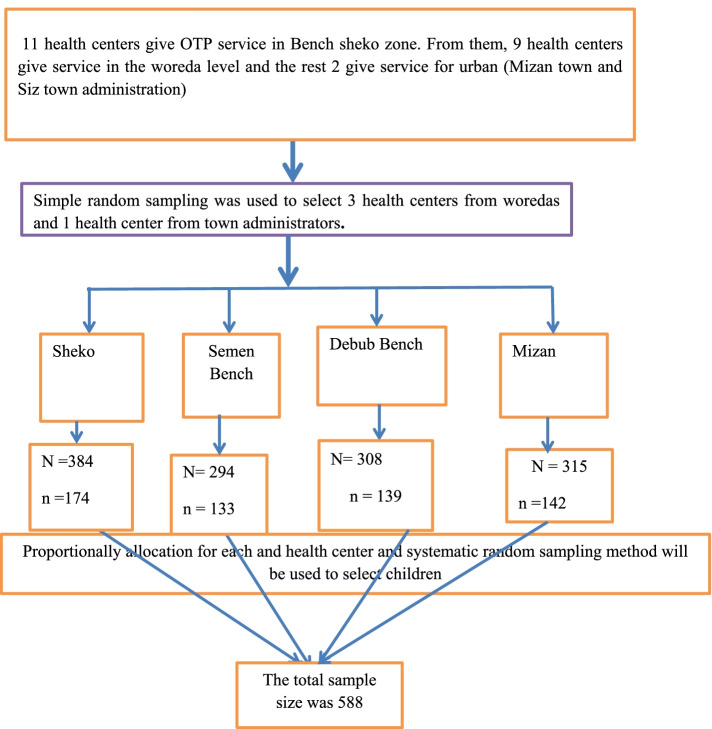
Shows the Sampling procedure among sever acutely malnourished 6–59 months of children treated with the outpatient therapeutic program in Bench sheko zone from September 01.2018 to August 30. 2019

In the study area there are a total of 11 health centers which are providing OTP service in Bench Sheko zone. Simple random sampling was used to select three health centers from woreda and one health centers from town administration. According to the data found from selected health centers total admission on the outpatient program between the years September 01, 2018, to August 30, 2019 was 2166. From those 214 were age greater than 59 months, no admission at age less than 6 months, 520 respondents had incomplete data, and transfer in and transfer out cases were 131. Out of 2166 respondent cards 1301 cards were eligible for the current study and 865 respondent cards were excluded from the study by using the exclusion criteria. Total eligible admission cards were listed in selected health centers. The total sample size is proportionally allocated to each health centers based on the total number of cards, finally cards were selected using simple random sampling technique (as shown Fig. 1).

## Characteristics of OTP

The risk period begins at the date when the child enters into the program and ends on the date when the child cure, dead and default from the program.

Censored consider as individual who have not recovered which mean it is either of defaulters, death, and Non- responder.

Cured/recovered- are those individuals who have become free from medical complications and have achieved and maintained sufficient weight gain and children under OTP treatment who discharged recovered or Weight-for-height/length Z-score is ≥ − 2 SD, and child has had or MUAC is ≥125 mm, with no edema for two consecutive weeks [11].

Readmission- patients that are declared cured or recovered but relapsed to be admitted to OTP.

Non-responder the patient did not meet discharge criteria after 8 weeks in treatment.

Defaulter- is a patient that is absent for two consecutive weeks and confirmed that the patient is not dead by home visit.

Ways of referral is how SAM child referred to use to OTP program; it may be self-refer (a mother herself), health extension worker (HEW), or the health development army.

Health extension workers are individuals who recruited based on nationally agreed criteria that include residence in the village, capacity to speak local language, graduation from 10th grade, and willingness to remain in the village to improve universal health care through health extension program [16].

Therapeutic finding refusal mean the child with SAM does not to take at least 75% of the recommended plumpy nut [17].

Admission status, it is the act of identification of the child whether he/she admitted before or new for the OTP program [18].

Plumpy nut (RUTF) high-calorie food given as a treatment for SAM children aged 6–59 months in the OTP program one sachet of plumpy nut is weighted 92 g and gives 500 kcal energy [17].

Routine medication is a drug given for SAM child who admitted in OTP to prevent hidden infection [5, 18, 19].

OTP evaluation, Children admitted at OTP evaluated every week to see the improvement from sever acute malnutrition, early detection of any medical complication during treatment, to give ready to use therapeutic feeding unit (RUTF) to eat at home and a course of routine medications including amoxicillin, vitamin-A, measles, and deworming. RUTF is a lipid peanut paste that resists bacterial contamination, contains very little water, does not require cooking, is energy dense, and meets the compositional requirement specified by them [20].

## Data processing and analysis

The collected data was edit, code, and enter into Epi data version 4.4.2 and then export to SPSS version 20 for windows program for analysis Exploratory data analysis was carried out to check the levels of missing values, presence of influential outliers, multi-collinearity, normality, and proportionality of hazards over time. The assumption for proportional hazard was assessed graphically by log minus log survival curve.

Cross tabulation, graph, and frequency tables were used to report the descriptive data. For comparison of survivor & estimate median duration of recovery on OTP, Kaplan Meir (KM) curve was used. The log-rank test was used to test whether the observed difference of recovery time between different groups of predictor variables was significant or not then proportional hazards Cox model with stepwise variable selection procedural were used to identify independent predictors of survival. Multivariable Cox proportional hazard regression analysis was carried out to identify predictor variables. Variables having a *P*-value ≤0.25 during bi- variable Cox proportional hazard regression analysis was entered into the multivariable analysis. Association was summarized by using AHR, statistical significance was declared at 95% CI, power 80% and P-value < 0.05*.*

## Data quality management

Data quality was maintained by recruiting data collectors who had taken CMAM training. The data collectors & supervisors were provided with intensive training for one day before data collection on the objective of the study and how to extract data for this study purpose, using data extraction format. The data collection process was closely monitored by the supervisors throughout the data collection period. Made close communication with data collectors and supervisors when gaps identified corrections were made timely.

## Results

### Socio-demographic/economic characteristics of study participants

A cohort of 588 children was followed retrospectively for a median time of recovery 49 days with an Interquartile range of 21 days. More than half 54.1% were female participants and 45.9% of the male. From total study participants, 320 were recovered from out of recovered 158 (49.4%) male children whereas 162(50.6%) were female. More than half of children admitted at OTP 65.6% went more than two hours to get a health facility. Nearly three fourth study 465 (79.1%) participants were from the rural area. More than half of the study participants 312(53.1%) were aged greater than 24 months and 276(46.9%) were age less than 24 months (Table 4).

**Table 4:** Socio-demographic and health service-related data of children in the outpatient therapeutic feeding program in Bench sheko zone, SNNP, Ethiopia from September 01, 201to August 30, 2019

Variables	Categories	Event to recovery		Total (%)
		recovered	Censored	
**Sex**	Male	158(49.4%)	112 (41.9%)	270 (45.9%)
	Female	162 (50.6%)	156 (58.1%)	318 (54.1%)
**Age the child in months**	< 24 months	143 (51.9%)	133 (43.6%)	276 (46.9%)
	> = 24 months	185 (56.4%)	127 (48.8%)	312 (53.1%)
**Residential area**	Urban	67 (20.4%)	56 (21.5%)	123 (20.9%)
	Rural	261 (79.6%)	204 (78.5%)	465 (79.1%)
**Distance to a health facility in hours**	< 2 h	116 (35.4%)	86 (33.1%)	202 (34.4%)
	> = 2 h	212 (64.6%)	174 (66.9%)	386 (65.6%)
**Referred by who**	Self –referred	186 (56.7%)	151 (58.1%)	337 (57.3%)
	Health extension worker	142 (43.3%)	109 (41.9%)	251 (42.7%)

### Medical characteristics of children at OTP program

In the current study 52.7 and 38.8%, children’s had a history of diarrhea and vomiting at admission respectively while children’s had a history of cough and malaria accounts for 50.3and 28.9% respectively. In the present study, 71.3 and 18.2% of children had taken amoxicillin and anti-malaria medication respectively while children had taken vitamin A and deworming were 39.5% and 335 (57%) respectively (Table 5).

**Table 5:** Medical characteristics of children in the outpatient therapeutic feeding program in Benchi Sheko zone, SNNP, Ethiopia from September 01, 2018, to August 30, 2019

Variables		Event to recovery		Total (%)
		Recovered	Censored	
**Diarrhea**	yes	149 (57.7%)	161 (37.7%)	310 (52.7%)
	no	109 (42.3%)	266 (62.3%)	278 (47.3%)
**Vomiting**	Yes	125 (38.1%)	103 (39.6%)	228 (38.8%)
	No	203 (61.9%)	157 (60.4%)	360 (61.2%)
**Cough**	Yes	145 (57.1%)	151 (45.2%)	296 (50.3%)
	No	109 (42.9%)	183 (54.8%)	292 (49.7%)
**Blood in stool**	Yes	52 (20.6%)	60 (17.8%)	112 (19.0%)
	No	200 (79.4%)	276 (82.2%)	476 (81.0%)
**Malaria**	Yes	70 (19.8%)	100 (39.4%)	170 (28.9%)
	No	284 (80.2%)	154 (59.6%)	425 (72.3%)
**Admission status**	New	252 (76.8%)	173 (66.5%)	438 (72.6%)
	Readmission	76 (23.2%)	87 (33.5%)	163 (27.7%)
**Therapeutic feeding refusal**	Yes	76 (23.2%)	37 (14.2%)	113 (19.3%)
	No	252 (76.8%)	223 (85.8%)	475 (80.8%)
**Oedema at admission**	Yes	134 (40.9%)	101 (38.8%)	235 (40.0%)
	No	194 (59.1%)	159 (61.2%)	353 (60.0%)
**Admission Criteria**	Oedema	122 (59.2%)	84 (40.8%)	206 (34.2%)
	MUAC< 11.5	164 (56.2)	128 (43.8%)	292 (65.8%)
	WFH <3SD	50 (47.6%)	55 (52.4%)	105 (17.4%)
**Type of SAM at admission**	Marasmus	133 (40.5%)	105 (40.4%)	238 (40.5%)
	Kwashiorkor	139 (42.4%)	87 (33.5%)	226 (38.4%)
	Marasmus- Kwashiorkor	56 (17.1%)	68 (26.2%)	124 (21.1%)
**Amoxicillin**	Yes	241 (73.5%)	178 (68.5%)	419 (71.3%)
	No	87 (26.5%)	82 (31.5%)	169 (28.7%)
**Vitamin A**	Yes	141 (43%)	91 (35%)	232 (39.5%)
	No	187 (57%)	169 (65%)	356 (59.5%)
**Anti-malaria**	Yes	54 (16.5%)	53 (20.4%)	107 (18.2%)
	No	274 (83.5%)	207 (79.6%)	481 (81.8%)
**Folic acid**	Yes	109 (33.2%)	74 (28.5%)	183 (31.1%)
	No	219 (66.8%)	186 (71.5%)	405 (68.9%)
**Deworming**	Yes	204 (62.2%)	131 (50.4%)	335 (57%)
	No	124 (37.8%)	129 (49.6%)	253 (43%)

### Time-to-recovery and treatment outcomes of children with SAM

**Fig. 2:**
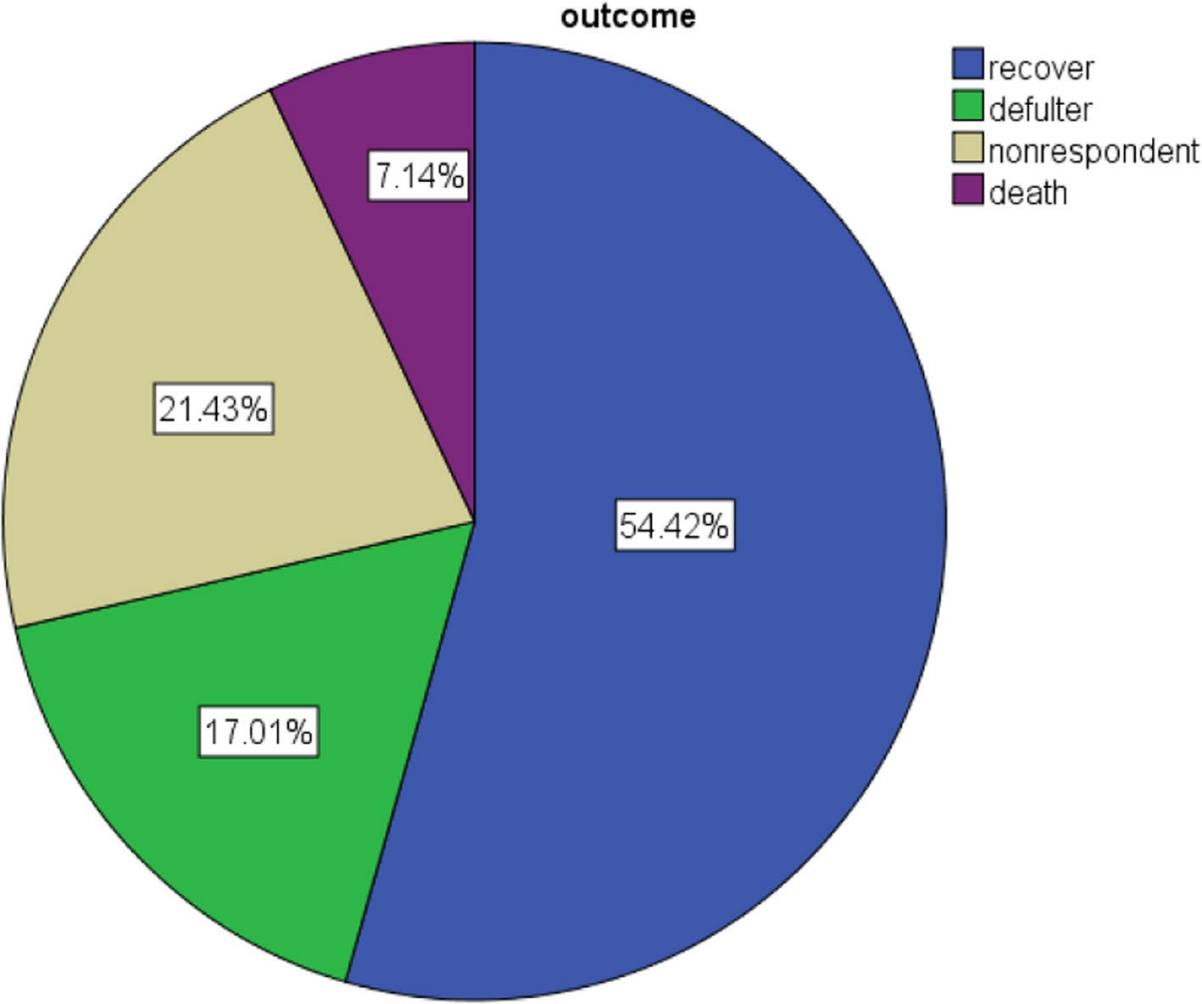
Treatment outcome of data of children in the outpatient therapeutic feeding program in Benchi sheko zone, SNNP, Ethiopia from September 01, 2018, to August 30, 2019

The present study 238 (40.5%) had been diagnosed as marasmus, 226(38.4%) had been diagnosed as Kwashiorkor and 124(21.1%) had been diagnosed as Marasmus- Kwashiorkor. From all study participants who severed acute malnourished (SAM) had been admitted at OTP in a selected health facility. The magnitude of recovery, defaulter, non-responder, and death frequencies are 320(54.4%), 100 (17%) 126(21.4%), 42(7%) respectively (Fig. 2). The study participants were followed for a total of 9408 person weeks and the incidence of the recovery rate of children admitted at the outpatient therapeutic program was 340 per 10,000 person weeks.

### Comparison of time to recovery among the different groups (the KM survival curve)

**Fig. 3:**
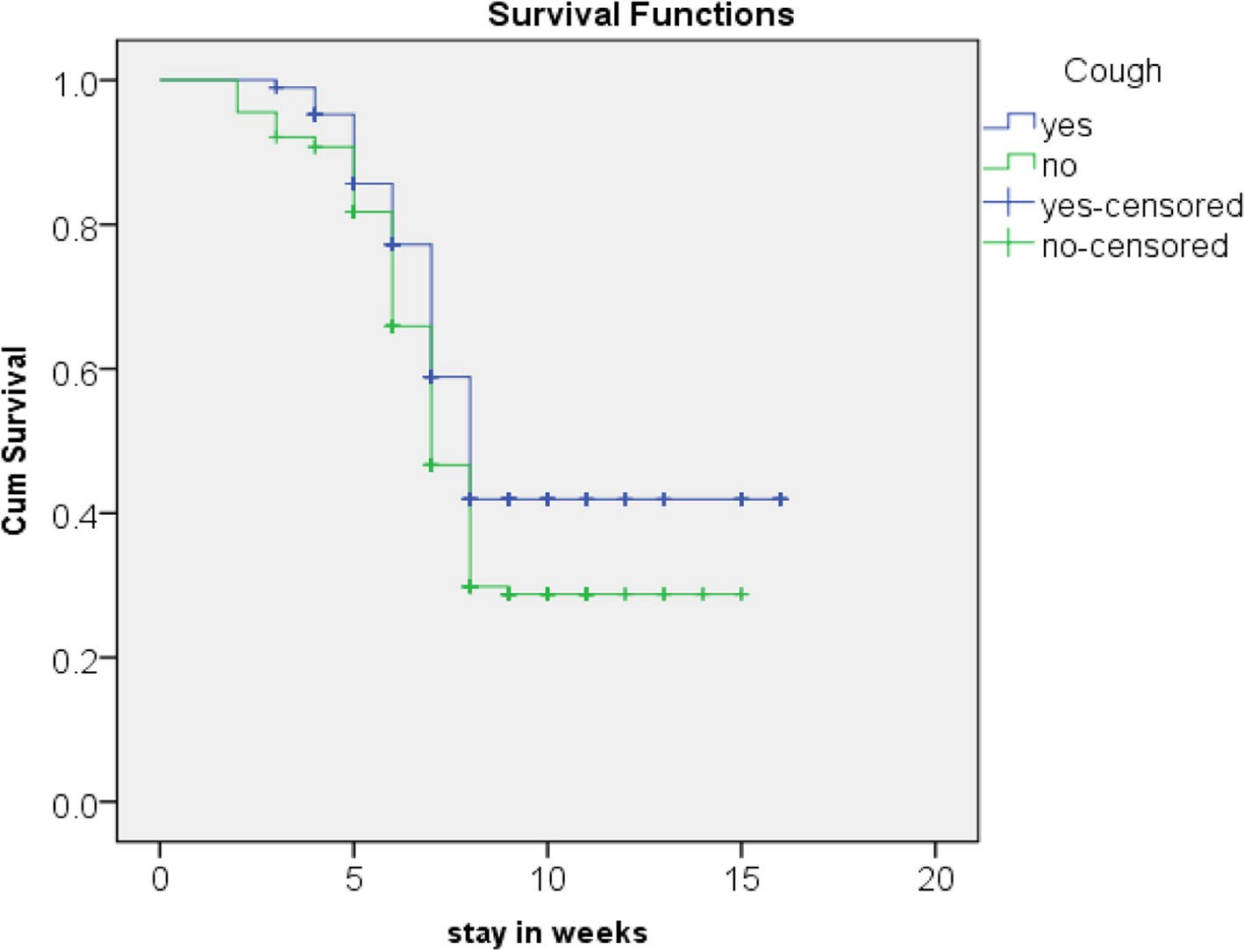
Kaplan Meir curve shows SAM children with cough and with no cough in the outpatient therapeutic feeding program in Benchi sheko zone, SNNP, Ethiopia from September 01, 2018, to August 30, 2019

The Kaplan Meir (KM) survival curve for cough estimates children’s free from cough had better cure rate and short median length of stay. The cure rate and median length of stay for cough free children were 62.8% and 49 ± 5 days respectively while children with history of cough were 49% and 56 ± 3 days respectively (*p* = 0.01) (as shown Fig. 3 and Table 6).

**Table 6:** Kaplan Meir comparison of median recovery time across different covariate; compare statistically using the log-rank test and present its P-value children’s admitted in the outpatient therapeutic feeding program in Benchi sheko zone, SNNP, Ethiopia from September 01, 2018 to August 30, 2019

Variable	Categories	Recovered	Censored	Log-rank	Medial time to recovery (weeks)	***p***-value
**Admission status**	New	252	173	15.550	7.89	< 0.001
	Readmission	76	87		8.74	
**Diarrhea**	Yes	149	161		8.63	
	No	109	166	31.465	7.61	< 0.001
**Cough**	Yes	145	151		8.45	
	No	109	183	11.39	7.78	0.001
**Bloody stool**	Yes	52	60		8.82	
	No	200	276	9.55	8.00	0.002
**Malaria**	Yes	70	100		9.00	
	No	154	264	30.55	7.83	< 0.001
**Deworming**	Yes	204	131	6.03	7.94	0.014
	No	124	129		8.33	

**Fig. 4:**
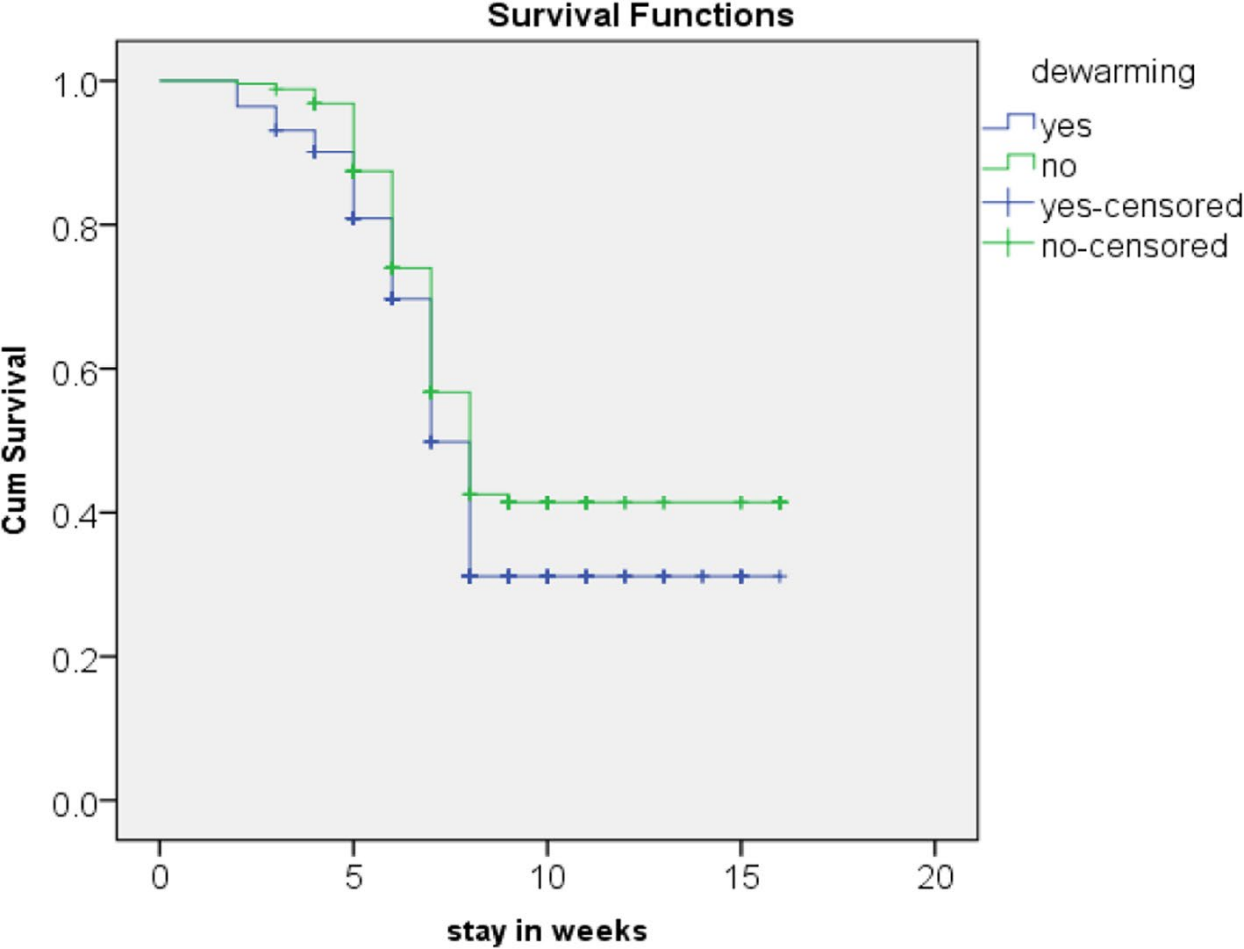
Kaplan Meir curve shows SAM children had taken deworming and had not taken deworming in the outpatient therapeutic feeding program in Benchi sheko zone, SNNP, Ethiopia from September 01, 2018, to August 30, 2019

The Kaplan Meir (KM) survival curve and life table for deworming estimates children’s take deworming had better cure rate and short median length of stay, accordingly. The cure rate and median length of stay were 204 (60.9%) and 49 ± 7 days respectively whereas children who had not taken deworming were (49.1%) and 56 ± 2 days respectively (*p* = 0.014) (as shown in Fig. 4 and Table 6).

### Factors associated with the recovery time of children on OTP

The following independent variables were candidates for the final model in the multivariable Cox regression analysis with *p*-value less than 0.25 which are, WFH, sex, feeding refusal, admission status, cough, diarrhea, malaria, and presence of blood in the stool, fever, presence of edema during admission, diagnosis at admission, treatment with amoxicillin, treatment with deworming, and treatment with vitamin A were forwarded in multiple variable analysis of proportional hazards Cox model.

### Cox proportional regression model showing predictors of recovery time

The rate of recovery from SAM among children who had been history of new admission status was 1.52 times higher than that of children had been history of readmission at any time during the study [AHR 1.52(95% CI: 1.17, 1.98)] . At any time during the study, the rate of recovery from SAM among children with no history of diarrhea were 1.9 times higher than those children with history of diarrhea [AHR 1.9 (95% CI: 1.52, 2.42)] . The rates of recovery from SAM among children with no cough were 1.4 times higher than those children with cough [AHR 1.4(95% CI: 1.13, 1.74)]. Children with no blood in stool at a point of time during the study had 1.55 times higher probability of getting recovered from SAM as compared to children with blood in stool [AHR 1.55(95% CI: 1.14, 2.10)] . The rate of recovery from SAM among children who had no history of malaria were 1.75 times higher than those children who had history of malaria at any time during the study [AHR 1.75 (95% CI: 1.32, 2.32)]. At any time during the study, the rate of recovery from SAM among children who had been took deworming were 1.4 times higher than children had not been took deworming [AHR 1.4 (95% CI: 1.01, 1.61)] (as shown Table 7).

**Table 7:** Proportional hazards Cox model multiple variable analyses of determinants of survival/treatment outcomes of OTP in Benchi sheko zone, SNN, Ethiopia, from September 01, 2018 to August 30, 2019

Variables	Categories	Recovered	censored	CHR (95% CI)	AHR (95% CI)	***p***-value
**Admission status**	New	252	173	1.67 (1.21,2.03)	1.52 (1.17, 1.98)	0.002*
	Readmission	76	87	1	1	
**Diarrhea**	Yes	149	161	1	1	
	No	109	166	1.41 (1.35,2.11)	1.9 (1.52, 2.42)	< 0.001**
**Cough**	Yes	145	151	1	1	
	No	109	183	1.61 (1.13,1.74)	1.4 (1.13, 1.74)	0.002*
**Bloody stool**	Yes	52	60	1	1	
	No	200	276	1.2 (1.12,2.04)	1.55 (1.14, 2.10)	< 0.001**
**Malaria**	Yes	70	100	1	1	
	No	154	264	1.2 (1.01, 2.31)	1.75 (1.32, 2.32)	< 0.001**
**Deworming**	Yes	204	131	1.62 (1.03,1.63)	1.4 (1.01, 1.61)	0.04*
	No	124	129	1	1	

*p*- Value**highly significant * significant

## Discussion

In the present study 328(54.4%) children were cured from malnutrition, the result is by far below the standard 75% recovery rate recommended by SPHERE in 2011 [21–24]. And also lower than the study done in Tigray region, Afar Regional State and Kamba District, in Ethiopia and Kitu Hospital, Kenya, reported 76.8, 67.7, 61.78 and 73.3% respectively [8–10, 13, 25].

In the current study area the defaulter, non-respondent, and death rate was highest that was 17.0, 21.4 and 7.1% respectively. The defaulter, non-respondent and death rates were highest when compared with a study conducted in Afar 6.3, 4.9 and 2.8% defaulters, non-responder and died rate respectively [26] and in Tigray, defaulter, and death rate were 13.85 and 3.02% respectively [18]. The possible reason for the cure rate difference is because outpatient therapeutic programs still do not deliver at the nearby health post and despite there are 28 health centers available in our study area only less than half of them [6, 27] give OTP services.

In the current study, the median recovery time is 56 days, which is twofold higher than 28 days which were reported by SPHERE recommendation [21, 28]. The finding is comparatively highest than the study done in Afar and Shebedino reported 44.15 and 50 days median recovery time respectively [12, 29, 30]. The possible explanation might be that in the study area all work burden including inside and outside homework loaded on females. Male partners have not contribution on child-rearing as well as work outside of the home due to this mother lacks time to nurse their sick child at home. These cause low rate of recovery, patients stay at the program for a long time and increase the median time of recovery. Those children with extended time to recovery from (SAM) have crises at different levels; for families, treated subjects, and the country level. Children with extending the time to recovery begin their lives at a marked disadvantage such as learning difficulties in school, earn less as adults, and face barriers to participation in their communities. Mothers also devoted their full time to SAM children and abandon the rest of the children in the household it also increases the vulnerability of other family members to SAM. In the country level, there is a direct cost for treatment of SAM child who admitted for a prolonged time at OTP indirect cost is that loss economic development due to lack of productive humanpower, direct allocation of money for the treatment of unrecovered children form SAM and its complication. Typically, children treated in the community at OTPs with SAM have a case fatality of less than 5%. Whilst if time to recovery is longer medical complication will be occur and treated as inpatients, because of severe infections including pneumonia, diarrhea and sepsis, have a reported case fatality of 10 to 40%.8 It is also clear that children with complicated SAM have a high ongoing risk of mortality after discharge from health facilities [31–33].

The current study found that the rate of recovery from OTP among newly admitted 1.52 times higher than that of the patient with history previous admission. This finding was consistent with Ethiopian SAM guideline 2017 reported that children who were declared cure from SAM and admitted again with the same case would stay at an outpatient program longer time than those newly admitted children [26, 34]. The possible explanation could be that patients with a history of new admission had not been medical complications but in case of patient with history previous admission, the cause of readmission may due to latent (hidden) infection which causes decrease rate of recovery.

According to the current study children with diarrhea is independently associated with rate of recovery; accordingly, children with no diarrhea were 1.9 times higher rate recovery than those children with diarrhea. This finding was consistent with the study conducted in Tigray reported that children with no diarrhea had 2.2 times higher probability of getting recovered from SAM as compared to the patients with diarrhea [23, 35]. The possible explanation could be that patient with diarrhea causes increases peristalsis movement of food in gastrointestinal system and decrease rate of absorption that exacerbate malnutrition. The other reason is children with diarrhea might have loss of appetite that causes increase malnutrition risk.

The present study found that rates of recovery from OTP among children with no cough were 1.4 times higher than those children with cough. This study aligned retrospective cohort done in Addis Ababa showed that children with no pneumonia had 2.22 times higher rate of recovery from severe acute malnutrition than those with children with pneumonia [36, 37]. The possible explanation could be that children with cough might not be eaten the recommended amount of plumpy nut as children with no cough due to loss of appetite and the child might also vomit while coughing.

According to the current study children’s no blood in stool was 1.55 times higher rate recovery than those children with blood in their stool. The possible explanation could be SAM patients with bloody diarrhea may suffer further complications like anemia and anemia associated complications negatively hamper the rate of the recovery process.

According to the current study rate of recovery from OTP among children had no history malaria was 1.75 times higher than those children had history of malaria. This finding is aligned with study done in Pawi northwest Ethiopia rivaled children with no malaria 1.54 times higher recovery rate from sever acute malnutrition than those with malaria [10, 38]. The possible explanation could be malaria increase body temperature, decrease appetite, causes anemia due to hemolysis and hypoglycemia all this factor decrease rate of recovery when compared with SAM patient with no malaria.

The present study found that the rate of recovery from OTP was 1.4 times higher deworm children’s than counterparts. This finding was consistence with Study conducted in Tigray region reported children who had taken deworming 1.95 times higher recovery rate than children not taken deworming [10, 39]. Due to the unhygienic environment many children in developing world had a diverse type of intestinal parasitosis thus children’s recommended to deworm children every six month in case of the resource-limited area this difficult to practice and children become susceptible to different helminthiasis. The worm has an effect on the patient like loss of appetite due to abdominal destination and anemia it causes a decrease rate of recovery.

## Limitation of the study

Since it is a retrospective study, it is difficult to add new variables in the study. Due to this, we were unable to explore important predictors such as maternal and paternal educational status, history of breastfeeding and maternal nutritional status.

## Conclusion

In the current study recovery rate of children with severe acute malnutrition admitted at OTPs were below the standard however, time to recovery were longer than standards. Cough fever, diarrhea, malaria and deworming and admission status of the patient were found independently associated with recovery time.

## Recommendations

The findings of this study confirm that patients with medical complications staying longer in the OTP center whereas children had taken deworming made shorten the time of recovery. So, to decrease the length of stay in OTP program health professionals have to detect medical complication actively and treat with appropriate antibiotics. Since the recovery/cure rate of children in the study are lower than the international SPHERE standard.

Health professionals and health extension workers have to early identification of illness and medical complication to treat with appropriate antibiotics. In addition health institution of the study area should be done cooperatively and arrange training opportunity for health professional on OTP. Governmental and non- governmental organizations should work with coalition to increase rate of recovery of children admitted at OTP.

## Data Availability

Data will be available upon reasonable request from the corresponding author.

## Abbreviations

AHR: Adjusted hazard ratio
BSHO: Bechi Sheko health office
CHR: Crude hazard ratio
CI: Confidence interval
GDP: Gross domestic production
IRB: Institute of review board
CMAM: Community-Based Management of Acute Malnutrition
EDHS: Ethiopian Demographic health survey
EPI-INFO: Epidemiological Information
FMOH: Federal Ministry of Health
HEW: Health Extension worker
Km: Kilometer
Mm: Millimeter
MOH: Minster of health
MUAC: Mid Upper Arm Circumference
SNNP: South nation nationality of people
OTP: Outpatient therapeutic program
PPS: Probability proportional to the size
RUTF: Ready-to-Use Therapeutic Food
SAM: Severe Acute Malnutrition
SD: Standard Deviation
SPHERE: Social and Public Health Economics Research Group
SPSS: Statistical package for social science
STATA: Statistics and data
UNICEF: United Nations Children’s Fund
UNSCN: United Nations System Standing Committee on Nutrition
WFH: Weight-for-Height
WHO: World Health Organization
